# Immunomodulatory nanodiamond aggregate-based platform for the treatment of rheumatoid arthritis

**DOI:** 10.1093/rb/rbz012

**Published:** 2019-04-19

**Authors:** Amanda Pentecost, Min Ju Kim, Sangmin Jeon, Young Ji Ko, Ick Chan Kwon, Yury Gogotsi, Kwangmeyung Kim, Kara L Spiller

**Affiliations:** 1 Department of Materials Science and Engineering, College of Engineering, Drexel University, Philadelphia, PA, USA; 2 School of Biomedical Engineering, Science, and Health Systems, Drexel University, Philadelphia, PA, USA; 3 KU-KIST Graduate School of Converging Science and Technology, Korea University, Seoul, South Korea; 4 Biomedical Research Institute, Center for Theragnosis, Korea Institute of Science and Technology, Seoul, South Korea

**Keywords:** drug delivery, nanobiomaterials, biomaterial–cell interaction

## Abstract

We previously demonstrated that octadecylamine-functionalized nanodiamond (ND-ODA) and dexamethasone (Dex)-adsorbed ND-ODA (ND-ODA–Dex) promoted anti-inflammatory and pro-regenerative behavior in human macrophages *in vitro*. In this study, we performed a pilot study to investigate if these immunomodulatory effects translate when used as a treatment for rheumatoid arthritis in mice. Following local injection in limbs of mice with collagen type II-induced arthritis, microcomputed tomography showed that mice treated with a low dose of ND-ODA and ND-ODA–Dex did not experience bone loss to the levels observed in non-treated arthritic controls. A low dose of ND-ODA and ND-ODA–Dex also reduced macrophage infiltration and expression of pro-inflammatory mediators iNOS and tumor necrosis factor-α compared to the arthritic control, while a high dose of ND-ODA increased expression of these markers. Overall, these results suggest that ND-ODA may be useful as an inherently immunomodulatory platform, and support the need for an in-depth study, especially with respect to the effects of dose.

## Introduction

Rheumatoid arthritis (RA) is a chronic inflammatory disease that affects over 24 million people worldwide [1]. It is characterized by the presence of excessive inflammation at the joints, which results in swelling, stiffness and constant pain. Currently, there are no treatments for RA that promote tissue regeneration.

On a cellular level, RA pathology stems from synovial infiltration and activation of a variety of immune cells, including macrophages, dendritic cells, mast cells, T cells and B cells [2]. Activated macrophages are one of the main producers of potent pro-inflammatory cytokines including tumor necrosis factor-α (TNF-α) [3], interleukin-1β (IL-1β) [4], interleukin-8 (IL-8) [5] and monocyte chemoattractant protein-1 [6], which perpetuate both the innate and adaptive immune response.[4] Macrophage production of these pro-inflammatory factors amplifies osteoclast function, resulting in increased bone resorption and loss during RA [7, 8]. Macrophage secretion of matrix metalloproteases also directly promotes extracellular matrix remodeling [9]. Thus, the extent of macrophage infiltration and activation corresponds directly with the RA inflammatory status, degree of surrounding destruction and overall prognosis [10, 11]. However, more recently it has become appreciated that depending on their phenotype, macrophages can act as crucial promoters of tissue regeneration [12].Therefore, an emerging field of research focuses on the design of biomaterials that can promote such pro-regenerative activities of macrophages [13–15].

One of the most potent treatments for RA is the administration of TNF inhibitors (e.g. adalimumab, infliximab). However, the strong immunosuppressive properties of these drugs lead to increased susceptibility to potentially fatal infections [16–18]. Also, macrophages secrete multiple pro-inflammatory mediators, not just TNF-α. An alternative treatment includes the administration of glucocorticoids (e.g. dexamethasone, Dex), which not only suppress production of multiple pro-inflammatory cytokines [19, 20], while stimulating the production of anti-inflammatory IL-10 [21], but also promotes pro-regenerative properties in macrophages such as phagocytosis of apoptotic cells [22] and regulation of extracellular matrix assembly [23]. Dex utilization by cells, however, is nonspecific, as glucocorticoid receptors are present in nearly all cells of the body [24]. In fact, Dex induces apoptosis in lymphocytes, which are key adaptive immune cells [21, 25]. Therefore, by increasing targeting of Dex to macrophages, these off-target effects may be mitigated. More preferably, Dex administration could be avoided altogether through the use of inherently anti-inflammatory biomaterials.

One strategy to target macrophages is through nano- and microparticles. Because macrophages are highly effective phagocytes, they naturally recognize and phagocytose nano- and microparticles upon introduction into the body [26]. Polymeric nanoparticles are one class of nanoparticles that are being investigated as Dex delivery vehicles. Recently, a study by Jia *et al*. [27] demonstrated that Dex-loaded liposomes significantly decreased production of TNF-α and IL-1β and reduced joint swelling in adjuvant-induced arthritic rats compared to free Dex. In another study, Wang *et al*. [28] reported that micelles loaded with Dex self-assembled with poly(ethylene glycol)-block–poly(ε-caprolactone) preferentially accumulated in the arthritic joints following intravenous administration and had local anti-inflammatory effects in adjuvant-induced arthritic rats. However, polymeric nanoparticles can induce complement activation, thus counteracting any anti-inflammatory effects of loaded drug by stimulating a pro-inflammatory response [28–30]. This significant negative effect indicates that there is a need for the development of nano- or microparticle platforms that are innately anti-inflammatory.

Nanodiamond (ND) is a commercially available carbon nanomaterial that is currently being investigated as a drug delivery vehicle because of its small primary particle size (∼5 nm), tunable surface chemistry, cytocompatibility with various cell types [31] and ability to be cleared from the body without causing liver damage [32–34]. Under physiological conditions, nanodiamond aggregates into micron-sized aggregates, which is the ideal size for uptake by macrophages without promoting pro-inflammatory activities [35]. In our previous study, both octadecylamine-functionalized nanodiamond (ND-ODA) (1–7 µm in diameter weakly bonded aggregates) and Dex-adsorbed ND-ODA (ND-ODA–Dex) aggregates (1–2 µm in diameter) demonstrated significant anti-inflammatory effects on primary human monocyte-derived macrophages *in vitro* [36]. Furthermore, ND-ODA, even without the addition of Dex, also increased the expression of CD163, a phenotype marker of macrophages that have been associated with tissue regeneration [37–40].

In this study, we first expanded on the characterization of the morphologies, structures and surface charges of ND-ODA and ND-ODA–Dex materials. Then, to determine if the previously observed immunomodulatory effects of these materials on macrophages translate *in vivo*, a pilot study was performed in which Dex, ND-ODA and ND-ODA–Dex were locally delivered to the arthritic limbs of mice with collagen type II-induced arthritis (CIA), a commonly used model for RA. The results highlight the potential for ND-ODA to be used as an inherently anti-inflammatory platform either alone or in conjunction with pro-regenerative therapeutics (e.g. Dex) to treat chronic inflammatory diseases, such as RA, and work to repair tissue damage.

## Materials and methods

### Experimental design

**Figure 1:**
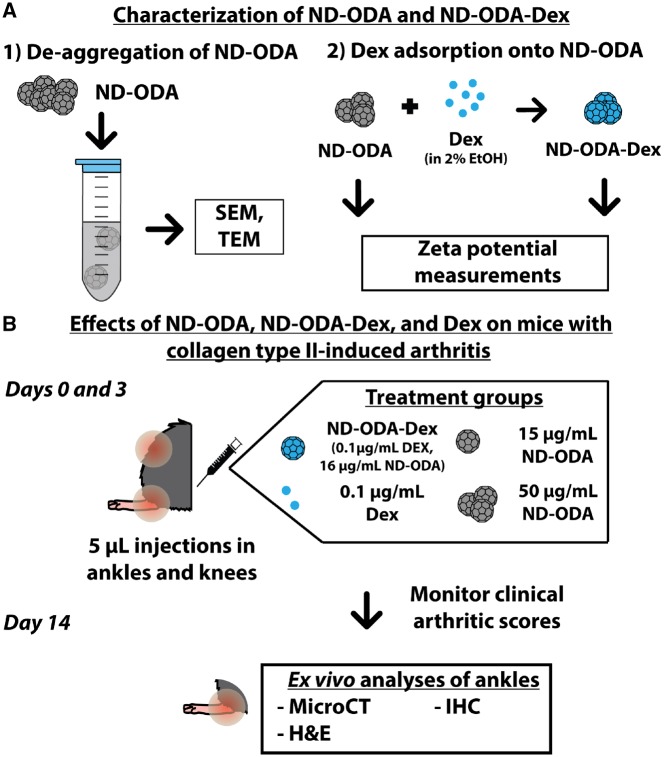
Overview of the experimental design. (**A**) Aggregation properties of ND-ODA were visualized using SEM and TEM for as-produced and bath sonicated filtered ND-ODA. Dex adsorption onto ND-ODA was also characterized for changes in surface charge via zeta potential measurements. (**B**) After preparing the collagen type II-induced mouse model, either Dex, ND-ODA (at low and high concentrations) or ND-ODA–Dex (at equivalent low ND-ODA and Dex concentrations) was injected locally in the arthritic knees and ankles. Three days later, a booster injection of the treatments was given. The arthritic clinical score was monitored during the 14 days following the initial injection. After 14 days, the arthritic hindlimbs were harvested, and *ex vivo* analyses (microCT, H&E and IHC) were performed

To complement our previous study [36], we performed additional characterization of ND-ODA and ND-ODA–Dex powders. First, the morphology and structure of as-produced ND-ODA and de-aggregated ND-ODA were visualized using scanning electron microscopy (SEM) and transmission electron microscopy (TEM) (Fig. 1A). Next, the change in surface charge was estimated by measuring the zeta potentials of both ND-ODA and ND-ODA–Dex. CIA, a commonly used RA model [41], was developed in male DBA/1J mice over the course of 3 weeks. After 3 weeks, a booster injection of collagen type II was given to the mice, and arthritis progression was monitored by assigning clinical arthritic scores. After the mice had developed moderate arthritis symptoms, their arthritic ankles and knees were injected with 5 μl of the prepared treatment, which consisted of Dex, ND-ODA (at low and high doses) or ND-ODA–Dex (Fig. 1B). After 3 days, the mice received a booster injection of the treatments. The mice continued to be clinically scored for an additional 11 days, after which they were sacrificed, and their arthritic ankles were harvested for *ex vivo* analyses.

Several endpoint analyses were performed to determine the effects of the treatments at both the tissue and cellular levels. First, X-ray micro-computed tomography (microCT) was used to visualize the bone structure from the whole arthritic ankles and assess changes in bone volume. Hematoxylin and eosin (H&E) staining was performed on paraffin-embedded tissue sections to determine the cell infiltration profiles. Finally, immunohistochemical (IHC) staining was performed to evaluate differences in the expression of F4/80, a pan-macrophage marker [42], as well as iNOS and TNF-α, two markers of pro-inflammatory macrophage behavior [43, 44].

### Synthesis and characterization of ND-ODA

Detailed description of the method for synthesizing ND-ODA can be found in our previous publications [36, 45]. Briefly, as-received UD90 grade ND powder with the primary particle size of ∼5 nm (sp3 Diamond Technologies) was oxidized in air and acid purified to reduce sp^2^-bonded carbon content, remove metal impurities and produce carboxylated ND (ND-COOH) [46]. ND-ODA was then synthesized through chlorination and subsequent reaction with octadecylamine. The resultant ND-ODA powder was rinsed in methanol to remove excess reactant [45]. The as-produced ND-ODA powders were sputter-coated with platinum and palladium and visualized using SEM at an operating voltage of 5 kV (Zeiss Supra 50VP, Germany). Large aggregates (>10 µm) were removed by dispersing the ND-ODA powder in deionized (DI) water, bath sonicating for 1 min, then filtering using a 10 µm cell strainer (pluriSelect). To discern changes in aggregate size, de-aggregated powders were visualized using TEM at an operating voltage of 200 kV (JEOL JEM-2100, Japan). To prepare the sample for TEM, the filtered ND-ODA solution was dropcast onto a lacey carbon-coated copper grid and allowed to air dry overnight. Because of ND-ODA’s strong tendency to aggregate in aqueous solutions [33], the aggregated ND-ODA powder could only be viewed under TEM, as the smaller aggregates’ adhesion to the fine grid aided in preventing re-aggregation. Aggregate and particle diameters were analysed using ImageJ software and are presented as mean ± standard deviation (SD).

### Synthesis and characterization of ND-ODA–Dex

Using the isotherm for Dex adsorption onto ND-ODA in a 2% ethanol aqueous environment [36], Dex was adsorbed onto ND-ODA such that the adsorption activity was ∼10 mg Dex per 1 g ND-ODA. To achieve this loading, individual samples were prepared by adding 3 mg ND-ODA to a 10 ml solution of 120 µg/ml Dex in 2% ethanol. After briefly bath sonicating and allowing to mix overnight, the Dex-adsorbed complexes (ND-ODA–Dex) were separated from unbound Dex by centrifuging at 3000 rpm for 2 h (Combi514R, Hanil Science Industrial, South Korea). The absorbance of the unbound Dex supernatant was measured at 242 nm using UV–visible spectrophotometry (UV-vis, Lambda 7, PerkinElmer, USA), and was related to the Dex concentration using a linear calibration curve generated at the same wavelength. To confirm adsorption capacity, the changes in Dex masses (mg), caused by Dex adsorption onto ND-ODA, were calculated for each individual sample, then divided by the masses of ND-ODA (g) that were used to adsorb Dex in each sample. Following separation, the ND-ODA–Dex complexes were freeze-dried overnight. To measure changes in zeta-potential, both ND-ODA and ND-ODA–Dex powders were dispersed in DI water and briefly bath sonicated prior to measurements (Malvern Zetasizer Nano ZS, USA).

### Development of collagen type II-induced arthritic mouse model

All experiments with live animals followed the relevant laws and institutional guidelines of the Institutional Animal Care and Use Committee in the Korea Institute of Science and Technology. The animals were subjected to a light cycle between 8 AM and 8 PM, and a dark cycle between 8 PM and 8 AM. The temperature was kept between 20 and 24°C and the humidity was kept between 40 and 60%. The animals were given UV sterilized water and food (Lab Diet 5053). Animal contact was allowed when providing food and water, as well as when the animals were being monitored and treated. CIA was developed in male DBA/1J mice of 4 weeks of age using bovine type II collagen and complete and incomplete Freund’s adjuvants according to the manufacturer’s protocol (Chondrex, USA). Briefly, equal parts of bovine type II collagen and complete Freund’s adjuvant were mixed and cooled on ice. The emulsion (100 µl) was injected intradermally into each mouse, about half an inch below the base of the tail. Three weeks later, a booster of bovine type II collagen emulsified with equal parts of incomplete Freund’s adjuvant was injected intradermally, at a slightly different location near the base of the tail. Mice were then monitored, and their paws were assigned clinical scores according to the severity of swelling and erythema [41]. The scores varied between 0 and 4, with 4 representing maximal redness and swelling and 0 representing normal, healthy appearance. After each mouse’s 4 paws had reached an average score of 10 (out of a possible 16), the mice were randomly grouped and treated.

### Local injection of mice with ND-ODA, ND-ODA–Dex and Dex

Mice that had developed arthritis were treated with ND-ODA, ND-ODA–Dex or Dex (*n* = 4–6 mice per treatment). Hindlimbs were injected with 15 or 50 μg/ml ND-ODA (referred to as low and high ND-ODA, respectively), ND-ODA–Dex (0.1 μg/ml Dex adsorbed onto 15 μg/ml ND-ODA) or 0.1 μg/ml Dex in PBS, at both the ankles and knees with 5 μl at each site. The ND-ODA and ND-ODA–Dex dispersions were prepared in PBS, followed by bath sonicating for 1 min and filtering through a 10 µm cell strainer, as described in our previous work [36]. To account for ∼70% mass loss due to filtering, high ND-ODA was initially prepared at 330 µg/ml, as previously described in [36]. Following bath sonication and filtering, the concentration was assumed to be ∼100 µg/ml ND-ODA. This dispersion was diluted to produce 15 µg/ml ND-ODA. The produced ND-ODA–Dex powders were pooled and the average weighted adsorption activity was calculated based on weight percent contributions. Using the average adsorption activity of ∼10 mg Dex/g ND-ODA and assuming 70% mass loss, 1 mg ND-ODA–Dex was dispersed in 30 ml PBS to produce a dispersion that was roughly comprised of 0.1 µg/ml Dex adsorbed onto 15 µg/ml ND-ODA. ND-ODA–Dex was dispersed in PBS and filtered immediately prior to injection to minimize Dex desorption.

Injections were performed using a Hamilton syringe, and the needle (32 gauge) was guided directly to the joint spaces at the ankles and knees. The mice were treated twice, once at day 5 and once at day 8 following the booster injection of collagen type II. Mice used as negative controls did not receive any injections. The arthritic scores were monitored over the course of 14 days. The data are presented as the mean total arthritic score ± SD. To discern the therapeutic effects at each timepoint between the experimental groups, a two-way ANOVA with Tukey’s *post hoc* multiple comparisons test was performed. The final average scores for the individual paws were also compared using a one-way ANOVA with Tukey’s *post hoc* multiple comparisons test. *P* < 0.05 was considered significant.

### *Ex vivo* microCT examination of arthritic hindlimbs

After 14 days, mice were sacrificed, and their arthritic limbs were excised and stored in 4% paraformaldehyde. Samples were randomly split into separate groups for microCT and immunohistochemical analyses, resulting in *n* = 2 replicates for each of the controls (because these controls have been extensively characterized in the literature [47–49]) and *n* = 4 for experimental groups in each analysis. The hindlimbs were analysed using a microCT imaging system (Quantum FX, PerkinElmer, USA). 3D images of the hindlimbs were reconstructed and utilized to determine bone and total limb volumes. The bone volume (%) was calculated by dividing the bone volume (mm^3^) by the total volume of the excised hindlimb (mm^3^) and multiplying by 100. Larger bone volume corresponds to less bone degradation. Bone volumes are represented as the average individual bone volumes ± SD. The low number of replicates in the control groups (*n* = 2) precluded statistical analysis of comparison to controls by either parametric or nonparametric tests.

### Histological examination

To proceed with histological and immunohistochemical analyses, the 4% paraformaldehyde was removed, and the treated and untreated arthritic hindlimbs were rinsed in running water for 5 min. The samples were then transferred to glass containers and decalcified for 24 h by submerging in Calci-Clear (National Diagnostics, USA). Next, the samples were rinsed and stored in PBS prior to paraffin-embedding and microtome sectioning to a thickness of 4 μm. Hindlimb tissue sections were deparaffinized by soaking in two rinses of xylene, each for 10 min, and then rehydrated in a reverse ethanol series. To visualize cell infiltration, the samples were stained with Harris’s hematoxylin and 1% alcoholic eosin Y (Sigma Aldrich, USA). The stains were differentiated by soaking in 1% acid alcohol for 20 s in between the two staining steps. Images were visualized in bright field using an EVOS optical microscope. At least 1–2 samples were analysed per group.

### Immunohistochemistry

Following deparaffinization and rehydration of hindlimb tissue sections, antigen retrieval was performed by immersing the tissue sections in 10 mM sodium citrate, and then heating to just below boiling for 20 min. The sections were then cooled and rinsed in running tap water. Next, endogenous peroxidases were blocked using the BLOXALL solution in the ImmPRESS Excel staining kit (Vector Laboratories, USA), followed by rinsing in a 10 mM sodium phosphate buffer solution (pH ∼ 7.5 in 0.9% PBS). 2.5% horse serum in PBS was then added to the sections for 20 min to block nonspecific binding. Polyclonal rabbit anti-mouse iNOS (ThermoFisher: PA3-030A, dilution 1:50 in 2.5% horse serum), polyclonal rabbit anti-mouse TNF-α (Abcam: ab34674, dilution 1:500 in 2.5% horse serum) or polyclonal rabbit anti-mouse F4/80 (Abcam: ab100790, dilution 1:50 in 2.5% horse serum) was added to the sections and incubated overnight at 4°C. Sections were washed twice in buffer solution for 5 min each, followed by the addition of the goat anti-rabbit IgG Amplifier Antibody (ImmPRESS Excel Staining Kit) for 15 min. Sections were then washed again in buffer solution for 5 min, and incubated with the horse anti-goat IgG ImmPRESS Excel Polymer Reagent for 30 min. Lastly, after 2 additional 5 min washes in buffer solution, the staining was visualized with 3,3′-diaminobenzidine (DAB) by mixing equal volumes of ImmPACT DAB EqV Chromogen Reagent 1 and Buffer Reagent 2 and adding it directly to the samples. To ensure consistency between samples, 100 μl of DAB was added to each section for 5 min to visualize iNOS and F4/80 staining and for 3 min to visualize TNF-α staining. In each batch of staining, a negative control was prepared by adding PBS instead of the primary antibody. These negative controls ensured the absence of strong nonspecific staining. Images were visualized in bright field using an EVOS optical microscope. At least 1–2 samples were analysed per group.

## Results

### Morphology of ND-ODA

**Figure 2:**
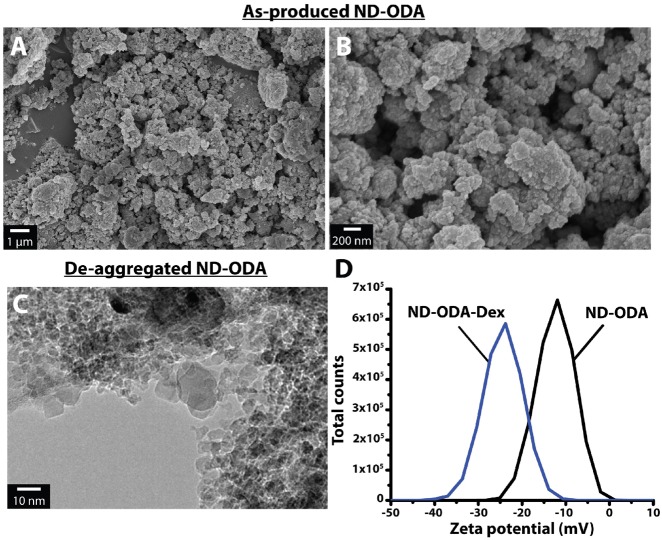
(**A** and **B**) SEM images of as-produced ND-ODA. (**C**) TEM image of ND-ODA de-aggregated via bath sonication and filtering through a 10 µm cell strainer. (**D**) Zeta potential measurements for ND-ODA–Dex and ND-ODA

The SEM images revealed the aggregation structure of as-produced ND-ODA. Smaller aggregates with an average diameter of 267 ± 95 nm amass to form larger aggregates with an average diameter of 1.9 ± 0.2 μm (Fig. 2A and B). These larger aggregates are densely packed, allowing for the formation of even larger, loosely bound agglomerates (>5 μm). In our previous work, we showed that bath sonication and filtering can break down these agglomerates such that the aggregate size is primarily within the 1–3 μm range [36], which is optimal for macrophage uptake [50]. The TEM images of de-aggregated ND-ODA confirm that these aggregates are made of individual ND-ODA particles that are 5.1 ± 2.2 nm in diameter (Fig. 2C).

### Zeta potential measurements of ND-ODA and ND-ODA–Dex

The average zeta potential of as-produced ND-ODA was measured to be −11.9 mV (Fig. 2D). The adsorption of Dex to form ND-ODA–Dex decreased the average zeta potential to −23.8 mV. Both zeta potential measurements had peaks in the −30 to +30 mV range, suggesting the potential for quick sedimentation of micron-sized aggregates in the dispersion.

### Effects of ND-ODA, ND-ODA–Dex and Dex on the clinical arthritic score of the hindlimbs

**Figure 3:**
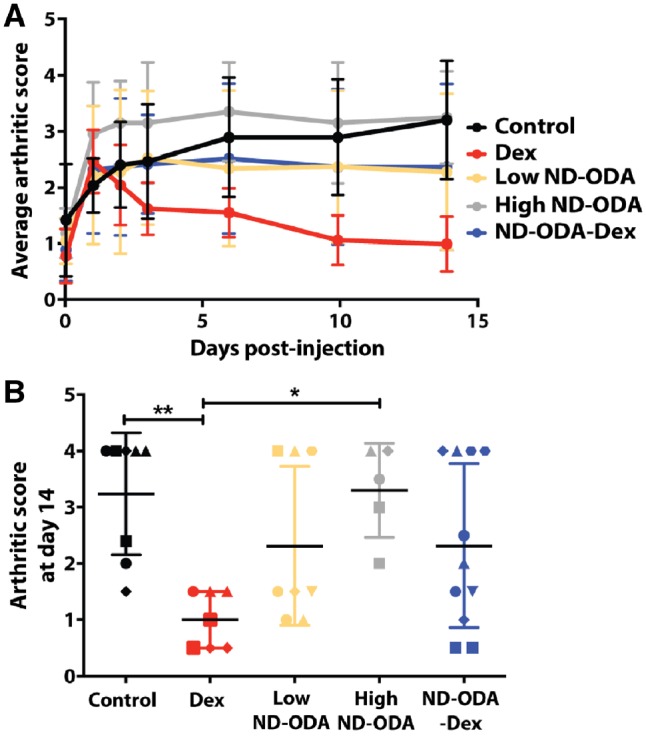
(**A**) Average arthritic score progression over time in response to Dex, low ND-ODA, high ND-ODA and ND-ODA–Dex treatments. The data are presented as mean ± SD. (**B**) Arthritic scores at day 14 shown as individual data points for each hindlimb. Different shapes represent different mice. Mean ± SD are also presented. *P < 0.05, **P < 0.01. For both analyses, *N* = 5–10 separate hindlimbs on 4–6 mice

Dex treatment resulted in a significant decrease in the arthritic score compared to the untreated CIA control mice as early as 10 days following the initial treatment (*P* < 0.01; Fig. 3A). In addition, starting on day 5, Dex treatment also resulted in significantly lower arthritic scores compared to the high ND-ODA treatment (*P* < 0.01), in agreement with previous results [48, 49]. Besides Dex, none of the other treatment groups had significant effects on the clinical arthritic scores over the course of 14 days. At the end of the study, Dex treatment resulted in significantly lower scores than both the CIA control (*P* < 0.01) and the high ND-ODA treatment group (*P* < 0.05; Fig. 3B). Notably, there was considerable variability within the low ND-ODA and ND-ODA–Dex treatment groups. Some samples resulted in very low scores approaching those of the Dex group, while others had very high scores on the order of the CIA control, resulting in nonsignificant decreases in average score compared to the CIA control.

### Effects of ND-ODA, ND-ODA–Dex and Dex on bone volume

**Figure 4:**
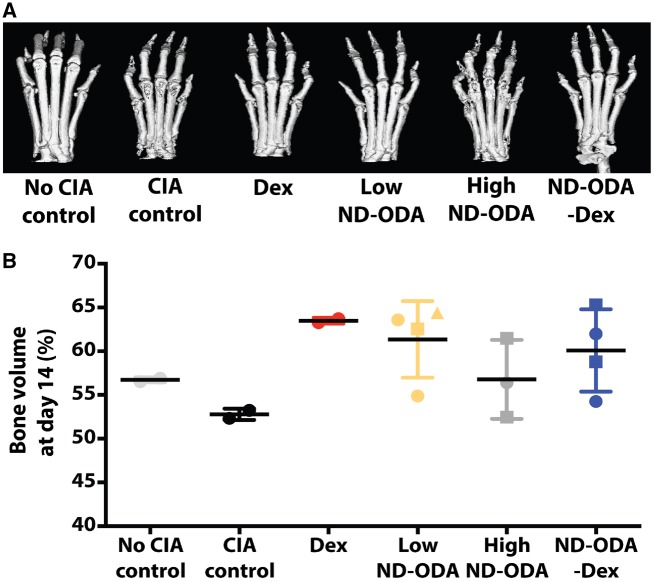
(**A**) Representative reconstructed microCT images of harvested arthritic limbs at day 14 post treatment. (**B**) Analysis of the average bone volume of the hindlimbs (*N* = 2 - 4 hindlimbs on 1–3 mice). Different shapes represent different mice. Data are presented as mean ± SD

From the reconstructed microCT images, Dex-, low ND-ODA- and ND-ODA–Dex-treated hindlimbs appeared to mitigate bone loss compared to the CIA control (Fig. 4A). Like the non-CIA control, their bone structures appeared to be smooth and continuous. However, in the CIA control and the high ND-ODA-treated hindlimbs, the bone structure appeared to be rough and patchy due to bone loss. Although statistical analysis could not be performed due to low numbers of replicates in the control groups (*n* = 2), it should be appreciated that all three ND-ODA treatment groups appeared to prevent bone loss, with low ND-ODA resulting in the highest bone volume (Fig. 4B).

### Effects of ND-ODA, ND-ODA–Dex and Dex on cell infiltration

**Figure 5:**
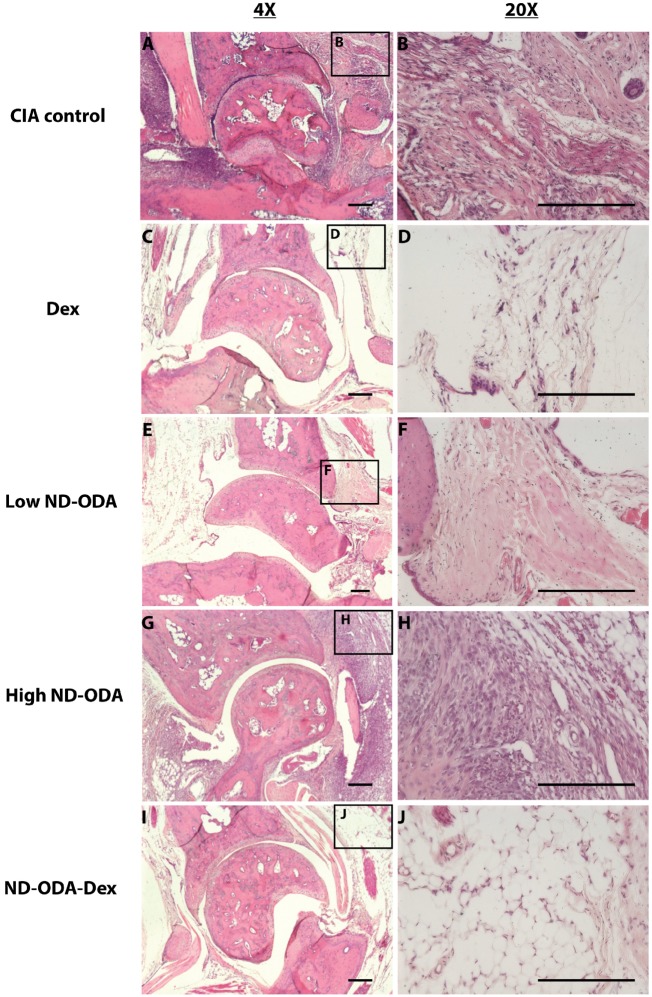
Representative images for hematoxylin (purple) and eosin (pink) staining of hindlimbs of CIA mice that were untreated (**A** and **B**), or treated with Dex (**C** and **D**), low ND-ODA (**E** and **F**), high ND-ODA (**G** and **H**) or ND-ODA–Dex (**I** and **J**). *N* = 2–3 hindlimbs on 1–3 mice. Images magnified at ×4 (left column) and ×20 (right column). The boxed sections represent the area that has been magnified. All scale bars are 200 μm

H&E staining showed that the CIA control had high levels of cell infiltration at the joint site (Fig. 5A and B). Likewise, the high ND-ODA treatment appeared to result in a large amount of cell infiltration (Fig. 5G and H). Dex-, low ND-ODA- and ND-ODA–Dex-treated joints appear to have reduced cell infiltration compared to the CIA control and to high ND-ODA treatment groups (Fig. 5C–F, I and J).

### Effects of ND-ODA, ND-ODA–Dex and Dex on macrophage presence and behavior

**Figure 6:**
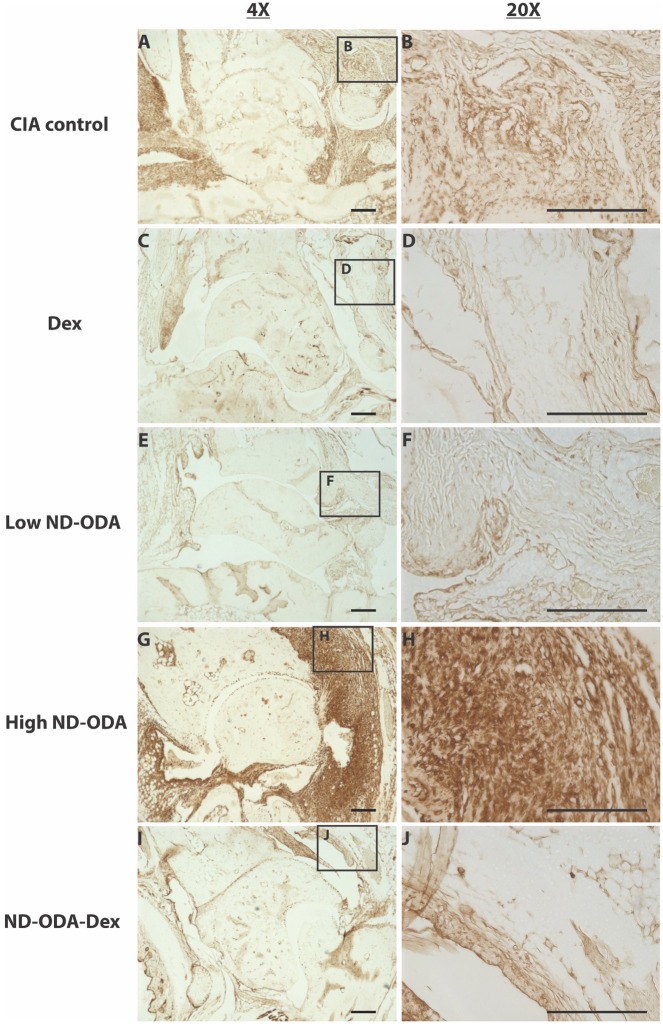
Representative images for immunohistological staining (brown) for F4/80 in the hindlimbs of CIA mice that were untreated (**A** and **B**), or treated with Dex (**C** and **D**), low ND-ODA (**E** and **F**), high ND-ODA (**G** and **H**) or ND-ODA–Dex (**I** and **J**). *N* = 2–3 hindlimbs on 1–3 mice. Images magnified at ×4 (left column) and ×20 (right column). The boxed sections represent the area that has been magnified. All scale bars are 200 μm

**Figure 7:**
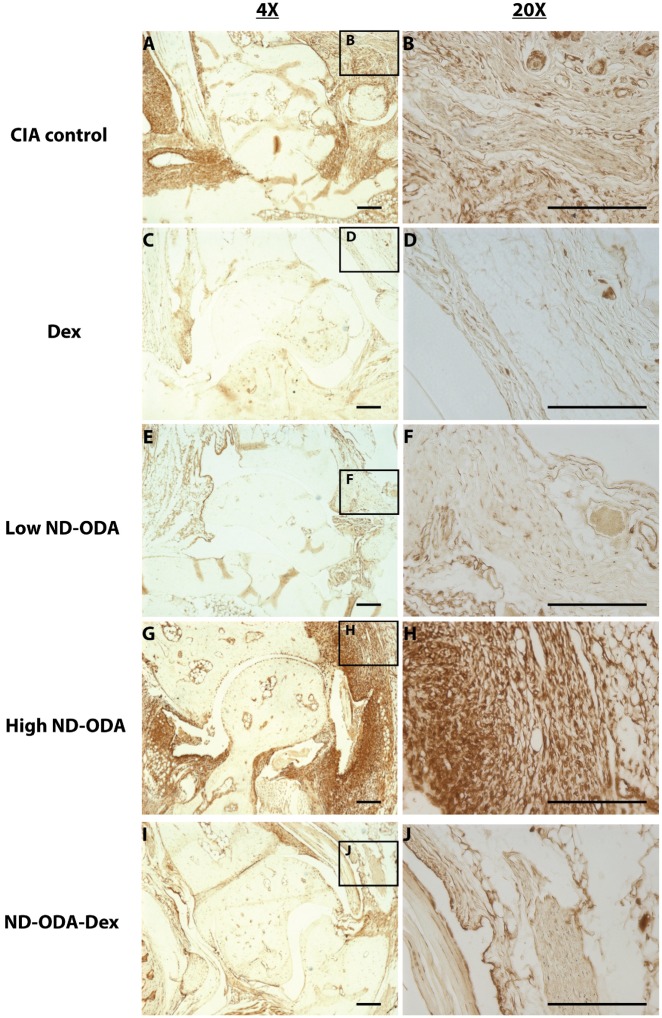
Representative images for immunohistological staining (brown) for iNOS in the hindlimbs of CIA mice that were untreated (**A** and **B**), or treated with Dex (**C** and **D**), low ND-ODA (**E** and **F**), high ND-ODA (**G** and **H**) or ND-ODA–Dex (**I** and **J**). *N* = 2–3 hindlimbs on 1–3 mice. Images magnified at ×4 (left column) and ×20 (right column). The boxed sections represent the area that has been magnified. All scale bars are 200 μm

**Figure 8:**
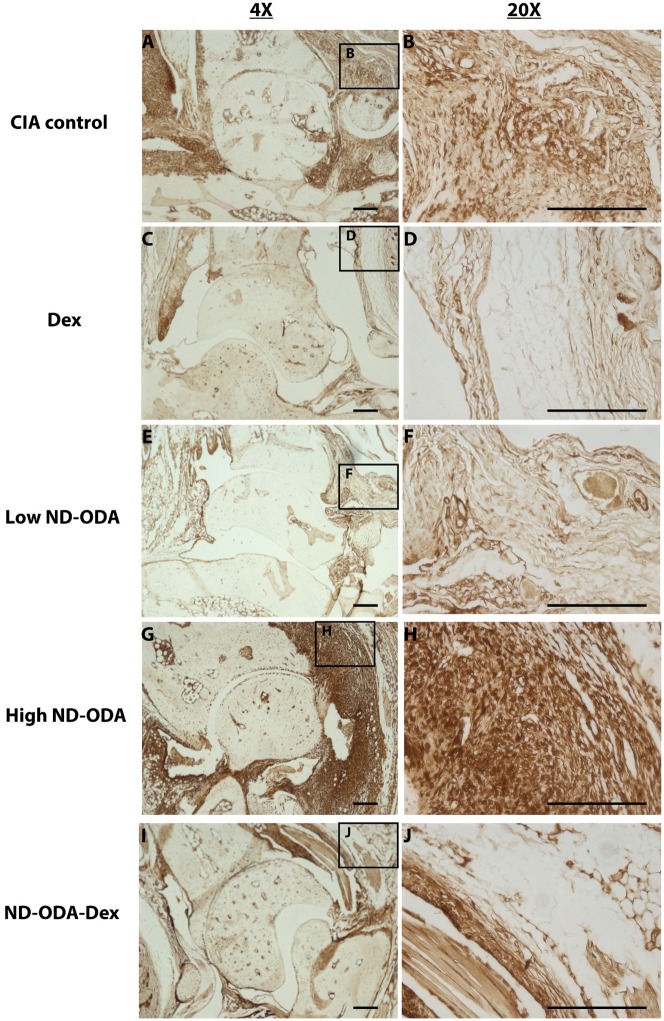
Representative images for immunohistological staining (brown) for TNF-α in the hindlimbs of CIA mice that were untreated (**A** and **B**), or treated with Dex (**C** and **D**), low ND-ODA (**E** and **F**), high ND-ODA (**G** and **H**) or ND-ODA–Dex (**I** and **J**). *N* = 2–3 hindlimbs on 1–3 mice. Images magnified at ×4 (left column) and ×20 (right column). The boxed sections represent the area that has been magnified. All scale bars are 200 μm

Expression of the pan-macrophage marker F4/80 as well as the pro-inflammatory markers iNOS, and TNF-α correlated with the cell infiltration profiles. Staining for each marker was darkest in the high ND-ODA-treated joints (Figs 6–8G and H), followed by the CIA control joints (Figs 6–8A and B). In contrast, joints treated with Dex, low ND-ODA and ND-ODA–Dex showed the lowest staining intensity (Figs 6–8C–F, I and J). Reduced F4/80 staining indicates that macrophages have decreased presence in the joint, while reduced iNOS and TNF-α staining indicate reduced inflammatory activity.

## Discussion

Overall, this study demonstrates that ND-ODA treatment may be able to target and modulate the behavior of immune cells, particularly macrophages and promote dose-dependent anti-inflammatory effects *in vivo*. This study expands upon our previous report, in which we demonstrated the anti-inflammatory effects of ND-ODA and ND-ODA–Dex on human macrophages *in vitro*. While preliminary, our results support our hypothesis that ND-ODA holds potential to be utilized directly as an inherently anti-inflammatory therapeutic to further promote the healing of damaged tissues caused by RA and other chronic inflammatory diseases. These results, together with the considerable variability in the clinical scoring results of the treatments, motivate the need for more in-depth *in vivo* investigations of ND-ODA, especially with respect to the effects of dose. In particular, future studies should investigate if ND-ODA (or ND-ODA–Dex) mitigates off-target effects associated with systemic Dex treatment.

Even without the attachment of Dex, our previous and current results indicate that ND-ODA has potential to be used as an inherently anti-inflammatory biomaterial, but only at the lower dose tested in this study. Using such materials may obviate the disadvantages provided by anti-inflammatory drugs, such as broad potent immunosuppressive effects, toxicity and drug resistance [16, 51, 52]. Furthermore, these materials can be used in conjunction with other regenerative therapeutics to augment their abilities to prevent and repair damaged tissues caused by RA and other chronic inflammatory disease progression. There are several strategies that can be implemented when designing immunomodulatory biomaterials, as biomaterial–macrophage interactions depend on a wide range of properties, including size, aspect ratio, surface chemistry, hydrophobicity, etc. [26, 53]. However, current research shows that the effects associated with these properties vary among material types, making it difficult to predict general trends that apply to all materials. For example, in one study by Nicolete *et al*. [54], poly(lactide-co-glycolide) (PLGA) 6.5 μm microparticles caused increased production of IL-1β and TNF-α in J774 murine macrophages when compared to 389 nm PLGA nanoparticles. However, in another study, Park *et al*. [55] reported that 20 nm silver nanoparticles induced the production of several pro-inflammatory cytokines in RAW 264.7 murine macrophages, compared to larger Ag nanoparticles. These seemingly contradictory results highlight the need for studying macrophage interactions with a wide range of materials to determine optimal properties for specific applications.

Although the results from this study and our previous study that suggest ND-ODA is inherently anti-inflammatory, there was significant variability within the treatment groups, especially in the clinical scoring data, suggesting that caution must be exercised before drawing definitive conclusions. The variability in the clinical scores may be due to: (i) actual variability in the anti-inflammatory effects of ND-ODA, including potentially pro-inflammatory effects that lessened the effects of Dex, (ii) the highly subjective and superficial nature of the scoring system, which has been reviewed elsewhere [56], (iii) variability in the CIA mouse model, which is highly sensitive to environmental stressors [57], (iv) difficulty in guiding the small, flexible Hamilton needle directly to the joint space and/or (v) difficulty in achieving uniform administration of ND dispersions in such small animal joints. In principle it is also possible that the materials were contaminated with endotoxin, which is difficult to measure when it is adsorbed to biomaterials [58]. However, we believe that endotoxin contamination is unlikely to explain the variability in the clinical scoring results because we used the same preparation methods that we used in our previous study in which we showed that these materials caused primary human macrophages to downregulate the potent pro-inflammatory markers TNF-α and IL-1β, which are both associated with the macrophage response to endotoxin [36]. Future research will be directed towards investigating all of these potential issues.

While our previous study of *in vitro* experiments with primary human macrophages showed that higher doses of ND-ODA increased anti-inflammatory activity compared to lower doses, our *in vivo* results suggested the opposite trend. In fact, the high ND-ODA treatment appeared to have pro-inflammatory effects, as evidenced by the strong presence of iNOS and TNF-α staining. Despite using the same low and high dose concentrations in both studies, it is likely that introduction into the joint changes the distribution of the particles and consequently the effective concentration exposure to macrophages and other cells. It is also possible that the high dose of ND-ODA has cytotoxic effects *in vivo*, although toxic effects were not observed with macrophages *in vitro* [36], considering that injured and dying cells release danger signals that would be expected to stimulate inflammatory behavior [59]. The interactions between macrophages and ND-ODA *in vivo* will be thoroughly evaluated in future studies.

Although our results indicate that a low dose of ND-ODA and ND-ODA–Dex may have anti-inflammatory effects *in vivo*, there were several limitations to this study. First, we could not determine statistical significance of experimental groups compared to CIA and Dex-treated controls because of the small sample size in the control groups. Because this was a pilot study, we chose to use a larger number of replicates for experimental groups compared to the well-studied control groups, but unfortunately this experimental design precluded statistical analysis. Nonetheless, the results of this pilot study do support the value of repeating the study with a larger number of animals. Another limitation is that tissues were only examined at the end of the study. To gain a better understanding of the interaction kinetics, future studies will examine the treated arthritic joints at several time points. The effects of varying the treatment, treatment dose concentrations and frequencies will also be explored since preliminary results suggest important dose-dependent effects. Finally, the functional effects of modulating macrophage behavior for tissue regeneration must be evaluated.

## Conclusions

Because of their proven anti-inflammatory effects *in vitro*, a preliminary *in vivo* study was conducted to evaluate the ability of ND-ODA and ND-ODA–Dex to target and modulate the behavior of macrophages in a murine model of RA. Our results suggest that low doses of ND-ODA and ND-ODA–Dex had some anti-inflammatory effects on the arthritic hindlimbs, although the results were variable within groups. These results support the need to conduct a more in-depth investigation to draw more definite conclusions regarding the anti-inflammatory activity of ND-ODA and ND-ODA–Dex *in vivo*.
